# Supplementary material for: A Low-Cost, Flexible Pressure Capacitor Sensor Using Polyurethane for Wireless Vehicle Detection
Source: Polymers (Basel). 2019 Jul 27;11(8):1247. doi: 10.3390/polym11081247 (PMC6723729; doi:10.3390/polym11081247)
Supplement: Supplementary file 1 [file polymers-11-01247-s001.zip › Supplementary files/Figure S1.doc]

Figure S1: (a) Sensor mounted on asphalt sample and putting into (b) Sample under test by Wheel-Track Device of Hamburg AASHTO T 324-04. (c) Recorded signal from tablet during device under test. (d) Illustration of testing operation.
